# Non-Hodgkin's lymphoma revealed by a cerebral vascular stroke: A case report

**DOI:** 10.1016/j.amsu.2022.103459

**Published:** 2022-03-03

**Authors:** Oufaa Jamal, Marouane Makhchoune, Yassin Tahrir, Khadija EL Guettabi, Abdelhakim Lakhdar

**Affiliations:** Neurosurgery Department, University Hospital Center IBN ROCHD, Casablanca, Morocco

**Keywords:** CEREBRAL LYMPHOMA, MRI, SURGERY, STROKE, Case report

## Abstract

Hemorrhagic lesions in CNS lymphoma are extremely rare. We report the case of a 75-year-old patient admitted to the emergency room following a classic hemorrhagic stroke. The CT scan showed a hyperdense tumor-like process with perilesional edema, the diagnosis reinforced by (MRI). The patient underwent macroscopically total surgical excision and the anatomo pathological examination concluded a diffuse non-Hodgkin's B large cell lymphoma. The follow up was marked by a clear clinical improvement. Primary cerebral lymphomas can be polymorphic, so this diagnosis should always be kept in mind during stroke manifestations. This case illustrates the diagnostic difficulty of this rare and poor prognosis condition.

## Highlights

• Primary central nervous system (CNS) lymphoma is relatively rare, representing only 1%–2% of all primary CNS malignancies. Hemorrhagic lesions in CNS lymphoma are extremely rare.
• This paper presents a case of A 75-year-old man admitted to the emergency room following a classic hemorrhagic stroke The CT scan showed a hyperdense tumor-like process with perilesional lesional edema, the diagnosis reinforced by (MRI). The patient underwent macroscopically total surgical excision and the anatomopathological examination concluded a diffuse non-Hodgkin's B large cell lymphoma. The follow up was marked by a clear clinical improvement.
• The clinical expression of primary cerebral lymphomas can be polymorphic, so this diagnosis should always be kept in mind during stroke manifestations. This case illustrates the diagnostic difficulty of this rare and poor prognosis condition.

## 1 Introduction

Primary central nervous system (CNS) lymphoma is relatively rare, representing only 1%–2% of all primary CNS malignancies. Hemorrhagic lesions in CNS lymphoma are extremely rare [1]. An abrupt, pseudo vascular onset is exceptional and most often reflects intra-tumor hemorrhage [1]. Here we report a case of 75 years old man admitted to the emergency room for a non-Hodgkin's lymphoma revealed by a cerebral vascular stroke.

## 2 Case report

A 75-year-old man patient, right handed, chronic smoker, living in a rural region was brought by his wife to the emergency room. He was presenting a rapidly progressive left hemi-body heaviness for 4 days, with a decline in general condition. There is no history suggestive of any mental or physical illness.

Neurological examination found conscious man with a left hemiparesis rate 3/5, hemihyposthesia, positive Babinski sign on the left, with a left facial paralysis of central aspect.

**Fig (1):**
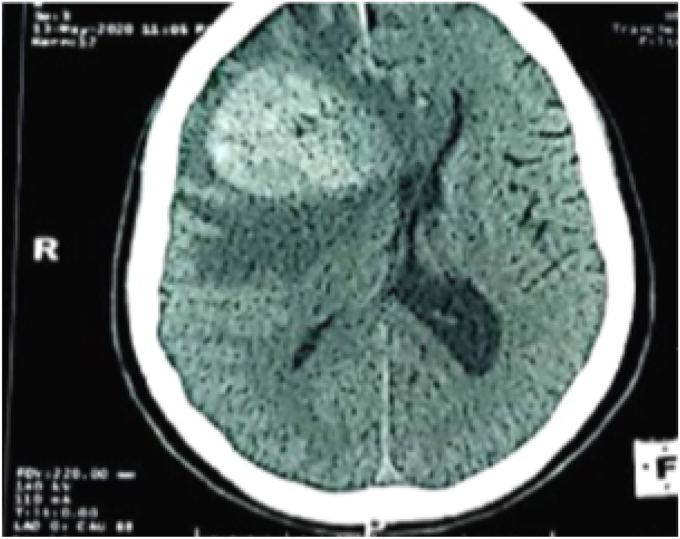
CT SCAN: right fronto-parietal formation that was spontaneously hyperdense and surrounded by a peri-lesional edema.

**Fig (2):**
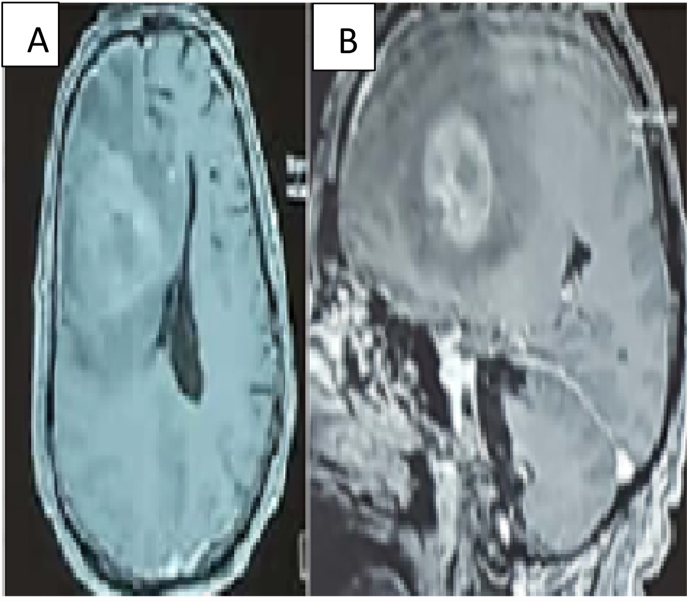
MRI: A: axial image, B: sagittal image right fronto-parietal formation surrounded by a peri-lesional edema. .

A CT scan The CT scan revealed a rounded, right fronto-parietal formation that was spontaneously hyperdense and surrounded by a peri-lesional edema, causing a big mass effect (Fig. 1).

we thought of a cerebral vascular stroke in front of the duration of evolution and the appearance on imaging but the presence of perilesional edema we complete by an (MRI). This last one reinforces the diagnosis of a tumoral process fig (2). The blood test was normal. We have retained the surgical indication.

The intervention was performed by our professor under general anesthesia. A wide frontoparietal craniotomy was performed after the dura was opened and corticotomy as well, we discovered a hematoma associated with a grey Aspirable tumour. A macroscopically total surgical excision was made without any incident.

The patient was extubeted the next day, the follow up was good persistence of the deficit on the left side. the histopathological exam concludes a diffuse non-Hodgkin's B malignant lymphoma with large cells.

**Fig (3):**
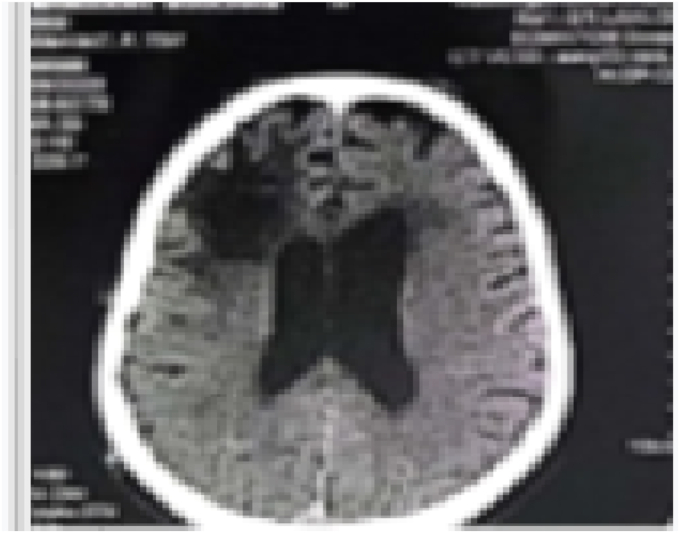
CT SCAN control 2 days after surgery no signs of tumor.

**Fig (4):**
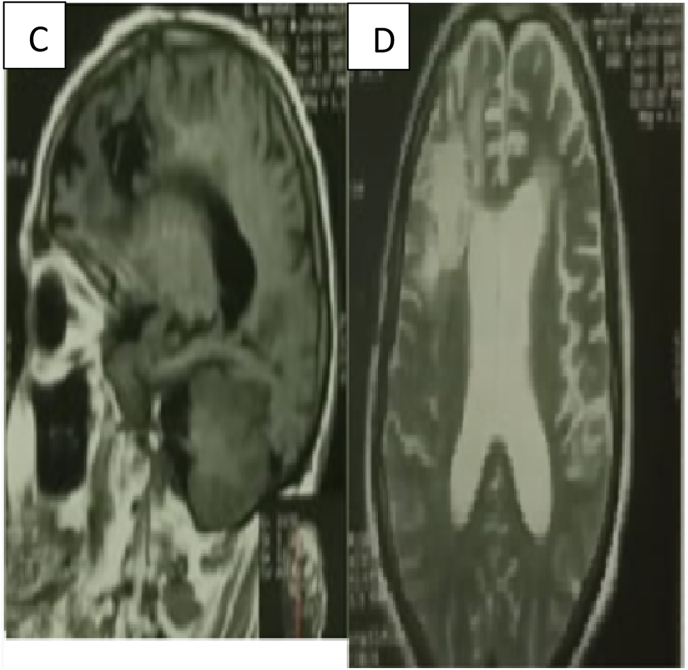
MRI: D: axial image T2, C: sagittal image. no signs of tumor.

The patient was placed on antiepileptic therapy. he was discharged from the hospital on the 6th day following and sent to the hematology department for management. At the third months during a control, a total recovery from his deficit the control imaging was satisfactory no tumor remains (Fig (3), Fig (4)).

This case has been reported in line with the 2020 SCARE guidelines [2].

## 3 Discussion

Brain tumors are not often involved in hemorrhage cerebral stroke and especially as the first sign of a tumor process, it is still much less frequent [1].

Hemorrhagic manifestations of lymphomas are extremely rare. In 70% of cases, there is a motor deficit, 43% neuropsychiatric signs, 33% signs of intracranial hypertension, 4% ocular symptoms and 14% seizures. The frequency of seizures is lower than for other brain tumors, due to more frequent invasion of deep structures. At diagnosis, 30%–50% of patients have an altered general condition, with a performance status (PS) of more than 2. During management, patients should have a thorough clinical examination, including neurological and ophthalmological.

Cognitive status should also be carefully assessed to document subsequent changes and possible sequelae [3]. Only 2 studies have reported [4,5]. Fukui et al. [4] reported a case of primary CNS lymphoma hemorrhage in a patient with AIDS, and Rubenstein et al. [5] in an immunocompetent patient. In these 2 studies, the Patients showed small amounts of intra-tumor hemorrhage Brain imaging by CT or MRI typically shows single or multiple deep periventricular lesions, taking contrast homogeneously. However, atypical presentations may simulate inflammatory diseases such as sarcoidosis or multiple sclerosis, acute demyelinating encephalomyelitis (ADEM) or other brain tumors (meningiomas, malignant gliomas, brain metastases). The radiological diagnosis is particularly difficult in the case of infiltrating lesions not enhanced by the product of Contrast, which occur in 10% of cases. In atypical presentations, MRI spectroscopy and perfusion sequences can provide arguments in favor of a cerebral localization of NHL, in our case the lesion was spontaneously hyperdense right fronto parietal with perilesional edema and slight contrast.

During surgery, we found a grayish aspirable lesion associated with the hematoma. This lesion was distinguished. Before surgery we were convinced that the lesion was a tumor and a tumor bleed, but it was surprising that the pathology at confirmation was a lymphoma [6].

Anatomo pathological examination remains essential, most often on a tumor specimen obtained by stereotactic biopsy. The brain biopsy can be avoided when lymphoma cells are found in the fluid Cerebrospinal fluid (CSF) [10–30% of cases] or in a glass sample [7].

Rubenstein et al. introduced an interesting immuno-histochemical result consisting of higher VEGF immunor-eactivity in the hemorrhagic case compared with 4 other primary CNS lymphomas that did not show hemorrhagic evidence [8].

On VEGF and hemorrhage in brain tumors, Arita et al. investigated the relationship between intratumoral hemorrhage and overexpression of VEGF in pituitary

adenomas, and they reported the positive relationship between VEGF expression and intratumoral hemorrhage in pituitary adenomas. Although the pathologies are different, their results on CD34 were similar to ours, and they also concluded that the amount of vascularity had no effect on hemorrhage [8]. We recently reported that the overexpression of VEGF and matrix metalloproteinases might play a role in metastatic brain tumor-associated hemorrhage. We also reported that one of the presumptive patho mechanisms could be through rapid growth and breakdown of vessels around the tumors caused by overexpression of VEGF and MMP from the tumor cells. Therefore, based on our own immunohistochemical results and

literature review, it is possible that hemorrhage of primary CNS lymphoma is related to VEGF activity [8].

Surgery has only a diagnostic role in this highly infiltrative, chemo and radiosensitive pathology. The therapeutic problems of PCL are multiple. In addition to the tumor-specific biological characteristics which seem to confer a poor prognosis to PCL in large B-cell NHL, the bioavailability of chemotherapeutics in the CNS is hampered by the BBB characterized by tight intercellular junctions, high Pgp expression and numerous cellular efflux mechanisms [7]. In addition, treatment-induced CNS toxicity is an essential limiting factor to be considered in therapeutic choices because of its potential severity, especially in the case of its potential severity, especially in the elderly (>60 years), who represent half of the patient population.

Although vascular manifestations are exceptional in PCL, this diagnosis must be suspected in front of recurrent neurological episodes of undetermined mechanism [9].

## 4 Conclusion

The clinical expression of primary cerebral lymphomas can be polymorphic, so this diagnosis should always be kept in mind during stroke manifestations. This case illustrates the diagnostic difficulty of this rare and poor prognosis condition.

## Ethical approval

Written informed consent for publication of their clinical details and/or clinical images was obtained from the patient.

Ethical approval has been exempted by our institution.

## Sources of funding

None.

## Author contribution

Oufaa JAMAL: writing the paper.

Marouane MAKHCHOUNE: writing the paper.

Yassine TAHRIR: Corresponding author and writing the paper.

Khadija EL GUETTABI: Correcting the paper.

Abdelhakim LAKHDAR: Correcting the paper.

## Research registration unique identifying number (UIN)

None.

## Guarantor

MAKHCHOUNE MAROUANE.

## Provenance and peer review

Provenance and peer review Not commissioned, externally peer-reviewed.

## Declaration of competing interest

The authors declare having no conflicts of interest for this article.
